# Practical Notes and Formulæ

**Published:** 1879-06-20

**Authors:** 


### PRACTICAL NOTES AND FORMULÆ.

**Remedies for Foul Breath.**—Dr. Howe's treatment varies, of course, with the cause. For foul breath from mental emotion he gives five or ten grains of musk several times a day, or one of the following recipes :

| | | |
|---|---|---|
| ℞ | Tinct. lavand. comp. | fl ℥ ij. |
| | Tinct. valerian, | fl ℥ ss. |
| | Mist. camph, | fl ʒ iij. |
| | Aquæ carui, | fl ʒ j. |

Dose, fifteen drops on sugar hourly until the mental disturbance ceases.

Or either of the following :

| | | |
|---|---|---|
| ℞ | Tinct. valer. ammon., | fl ʒ ss. |
| | Tinct. castorii comp., | fl ʒ j. |
| | Ætheris | gtts xv. |
| | Aquæ menthi, | fl ℥ jss. |

Half to be taken two or three times a day.

| | | |
|---|---|---|
| ℞ | Tinct. assafœtida | ℥ j. |
| | Tinct. hyosciami, | fl ʒ ij. |
| | Tinct. cinnam., | fl ℥ jss. |
| | Aquæ menth pip., | fl ʒ ij. |

Dose, one teaspoonful in water every three hours.

The following is to be given if the breath remains affected :

| | | |
|---|---|---|
| ℞ | Pulv. cinnam., pulv. pimentæ, pulv. cardom | aa ʒ ss. |
| | Sacchar. alb., | ʒ j. |
| | Mucilag. gum. acaciæ | q. s. |

Make fifty pills, and take as may be necessary. Fifteen drops of oil of nutmeg mixed in a teaspoonful of olive oil, to be rubbed on the gums, the author commends as a deodorizer. For bad breath from constipation, exercise, proper food, laxatives and deodorizers are prescribed—also the chewing of calamus root. For the diseased breath from dyspepsia the usual remedies for dyspepsia, and deodorizers are to be given. Congenital bad breath, Dr. Howe says, can only be palliated, not cured.—*Med. News.*

**Worms.**—A few weeks since I gave Mrs. C. the following mixture as a vermifuge:

| | | |
|---|---|---|
| ℞ | Santonini, | grs. xvj. |
| | Spigelii Ext. Fl., | gtts clx. |
| | Syr. simp., | ℥ ij. |

M. S g.—Teaspoonful morning and night.

She gave it about equally between four children of her own, and one of the neighbor's children. The result was the expulsion of sixty-seven long-worms. As having a possible bearing upon the question whether the worms cause any special symptoms by their presence in the intestines, allow me to say that the child for which the vermifuge was particularly desired, had previously to taking it, several attacks of convulsions. They ceased with the expulsion of the worms—*Med. Brief.*

**Dysentery and Cholera Infantum.**—The season having arrived for those summer complaints so annoying to the practitioner, we append a few favorite formulas, recommended for their treatment, some of which are reproduced from former volumes of our journal for the benefit of those who were not then subscribers.

℞. Corrosive sublimate..........1 gr.
Water..........1 pint.

Dose for Dysentery in an adult, from a half to one teaspoonful every three to four hours, until the action of the liver is fully restored, when with, or without an opiate, the case recovers, usually in three or four days. Salivation is less liable to take place than from the use of other forms of mercury.

**Chloral in Dysentery.**—Chloral, five to ten grains, by enema in two ounces of starch gruel, repeated as often as necessary, has been found very effectual in obstinate cases of dysentery, the patient being promptly relieved from the most distressing tormina, generally sleeping from three to six hours, and awaking refreshed and improved. In most cases it would be advisable before using this agent to give a purgative of salts or castor oil that all offending matter may be removed, before the checking up of the bowels by the enema. The slight burning with desire to evacuate which results from the injection is but temporary, and is soon followed by a sense of comfort and sound sleep.

**Sulphate of Magnesia in Dysentery.**—A dessertspoonful of epsom salts in a glass of water, with one grain of morphine, or a teaspoonful of paregoric, constitutes a favorite remedy for dysentery. It should be given in doses of a tablespoonful every one to two hours, until discharges are changed, and then not so frequently. Given early in the attack—the patient keeping quiet, and living on rice and boiled milk or other unirritating diet, taken sparingly; this remedy is among the best known to the profession for dysentery, and is coming into general use.

**Pepsin and Creosote in Cholera Infantum.**

℞. Sacharated pepsin..........gr xvj.
Creosote..........gtt ij.
Aquæ cinnamon..........
Glycerin..........aa ℥ ss.
M. Dose, half to one teaspoonful every three hours.

**Belladonna in Dysentery.**—Belladonna in doses of two to four drops of the fluid extract, every two to four hours until the griping pains and tenesmus are relieved, and then in gradually diminished doses, has been recommended as very efficient in dysentery. In some cases it is necessary to push the remedy until its constitutional symptoms are slightly felt before its full remedial effect is manifest.

**Subnitrate of Bismuth in Cholera Infantum.**

℞. Subnitrate of bismuth..........1 to 2 grs.
Pepsin..........1 to 2 grs.
Tannin..........½ to 2 grs.
Pulv. Cinnamon..........2 grs.
As a dose every two to three hours.

**Bromide of Potash in Cholera Infantum.**—Bromide of potass. in one to two grain doses given in mucilage of gum arabic every two to three hours, is a useful remedy in cholera infantum, and in the various forms of summer diarrhœa of children. Other treatment should be conjoined, or an occasional dose of chalk and mercury, and when fever exists, frequent small doses of aconite or gelsemium, with revulsive to bowels, etc.

When great thirst exists, it is usually advised to give frequent small quantities of ice-water. We have found it exceedingly delightful and comforting to the child in some cases to give it good fresh water to drink *ad libitum*, and we believe that *vast numbers of infants have died by withholding in this disease nature's remedy for thirst, pure cold water.* W.

IODOFORM is strongly recommended by Prof. Zeissel, of Vienna, as an internal remedy in syphilitic neuralgia. He relates two cases which resisted all other treatment and yielded only to this remedy—*Medical Times and Gazette.*

**Gargle for Angina.**—

℞ Carbolic acid,..........  ..................... .
Tannin,...... ......each......... ............ 15 parts.
Alcohol,... ........... ........................ 60 parts.
Distilled water......................................120 parts.
M. Sig. A teaspoonful in half a glass of water, once a day.

This gargle is greatly employed in Russia and in the Russian colony at Nice. It is of great service in commencing angina, and in all chronic irritations of the throat.—*Rev. Med.*

**Solvent for Salicylic Acid.**—

℞ Salicylic acid...... ..........................................dr. ij.
Sol. of acetate of ammonia...... .................oz. ij.
And of water...................................................oz. vj.
M. One ounce of this solution contains fifteen grains.

**Removal of Moles.**—Moles may be removed by the application of nitric acid, or by the use of the acid nitrate of mercury. Little or no pain results from the latter remedy. The sound skin should be avoided. The mole shrivels away and comes off in a few days, leaving a slight scar.

**Coryza.**—Rudolpho Rudolphi recommends the use of eucalyptus globulus for the rapid cure of *acute* coryza, or cold in the head. He has found, by numerous trials on himself and patients, that after chewing a few of the dried leaves and slowly swallowing the saliva, the affection is promptly relieved, often disappearing in the course of half an hour. The remedy is useful in acute cases only.—*Gazz. Med. Ital. Lombardia.*

**Toothache.**—Collodion, mixed with enough carbolic acid to form a jelly like mass, (about equal parts,) taken on the end of a stick and placed in the cavity of the tooth, is said by Mr. C. A. Guild, in the *Clinic*, to relieve the pain almost instantly, if it depends on an exposed nerve.

**Rapid and Certain Treatment of Simple Hiccough.**—Dr. Grelletty once saw a mother as tender, as full of affection for her children, give them a morsel of sugar dipped in table vinegar whenever immoderate or too rapid repletion of the stomach, or any other cause, had induced hiccough. The latter ceased as if by magic. Since then the Vichy physician has very frequently employed this means of his own account, and has never found it without avail.—*Ib.*

**Salicylic Acid Mixture.**—Prof. J. M. Dacosta gives the following formula for administering salicylic acid in solution, as being the most elegant and pleasant combination he has been able to devise.

| | |
|---|---|
| Sodii salicylat | 20 grains. |
| Spiritus lavendulæ | 15 drops. |
| Glycerinæ | 1 drachm. |
| Aquæ | 1 ounce. |

Mix.. Sig. One dose.—*Richmond and Louisville Med. Journal.*

**Dangers of Vulcanized Rubber Nipples.**—Dr. Forestier, of Lyons, reports two cases of poisoning in young infants brought up by hand, both of which were probably due to the employment of white vulcanized rubber nipples. The symptoms were analogous to those of poisoning by the sulphide of carbon, and as that substance is employed in the vulcanization of the rubber, it was in all probability the cause of the accidents. One of the cases terminated fatally.—*Physician and Pharmacist.*

**Cholera Infantum—Monobromated Camphor in.**—Monobromated camphor triturated with sugar, or sugar of milk, in the proportion of 1 grain to 10 of the sugar, is a good remedy for diarrhœa and cholera infantum; dose from one to two grains every two to three hours, for a child from 6 months to one year old. If rejected by the stomach, may be injected into the rectum with a little starch water.

**Emulsio Expectorans.**—

| | | |
|---|---|---|
| ℞ | Morphiæ sulph | grs. 16. |
| | Syr. scillæ | |
| | " ipecac | aa fl ℥ 16. |
| | " tolut | |
| | " pruni virg | aa fl ℥ 12. |
| | Tinc. benz. co | |
| | " sanguinariæ | aa fl ℥ 4. |
| | Aquæ | fl ℥ 6. |

Dose, a teaspoonful.

**Mistura Bronchi.**—

| | | |
|---|---|---|
| ℞ | Ammonii carbon | grs 10 |
| | Syr. ipecac | fl ʒ 1½ |
| | Tinc. opii camph | fl ʒ 1 |
| | Syr. pruni virg | fl ʒ ½ |
| | Aquæ q. s. ad | fl ℥ 2 |

Dose, a teaspoonful for children.

CASTOR OIL mixed with an equal weight of tallow or other oil is an excellent dressing for leather. Besides this, neither rats, cockroaches, nor other vermin, will attack leather so prepared.

